# Performing Multilingual Analysis With Linguistic Inquiry and Word Count 2015 (LIWC2015). An Equivalence Study of Four Languages

**DOI:** 10.3389/fpsyg.2021.570568

**Published:** 2021-07-12

**Authors:** Diana Paula Dudău, Florin Alin Sava

**Affiliations:** Department of Psychology, West University of Timisoara, Timisoara, Romania

**Keywords:** multilingual analysis, content analyses, automatic text analysis, Linguistic Inquiry and Word Count, LIWC, LIWC2015

## Abstract

Today, there is a range of computer-aided techniques to convert text into data. However, they convey not only strengths but also vulnerabilities compared to traditional content analysis. One of the challenges that have gained increasing attention is performing automatic language analysis to make sound inferences in a multilingual assessment setting. The current study is the first to test the equivalence of multiple versions of one of the most appealing and widely used lexicon-based tools worldwide, Linguistic Inquiry and Word Count 2015 (LIWC2015). For this purpose, we employed supervised learning in a classification problem and computed Pearson's correlations and intraclass correlation coefficients on a large corpus of parallel texts in English, Dutch, Brazilian Portuguese, and Romanian. Our findings suggested that LIWC2015 is a valuable tool for multilingual analysis, but within-language standardization is needed when the aim is to analyze texts sourced from different languages.

## Introduction

Within a short period, the *Internet of Things* made online communication vital for our lives in society. As the repository of psychologically relevant written language expanded massively at an accelerating pace, opening new possibilities for social science research worldwide, a pressure to automatize content analysis also arose (e.g., Shayaa et al., 2018). Content analysis means any systematic transformation of a string of text into statistically manageable data representing the presence, intensity, or frequency of some relevant features (Shapiro and Markoff, 1997). By extension, automatic content analysis refers to any transformation of such kind that is not performed manually by human raters but with specialized software or programming languages.

Linguistic Inquiry and Word Count 2015 (LIWC2015; Pennebaker et al., 2015) is a closed-vocabulary approach tool well-suited for the needs of psychologists with no or limited background in data science (Kern et al., 2016). Following a simplistic working principle, the tool provides any researcher with an automated, objective method for extracting insights about the attentional focus reflected through language (Boyd and Schwartz, 2021). More precisely, it consists of an internal dictionary and a piece of software designed for tokenization and word counting. Each word or word stem in the dictionary belongs to one or more pre-established categories with different meanings, most of them ensuing from psychological theories. The software scans the input text, makes a word-by-word comparison with the dictionary, and computes the percentage of words found in each category.

The history of Linguistic Inquiry and Word Count (LIWC) began in the early 1990s. Since then, two revised versions have been launched: LIWC2001 (Pennebaker et al., 2001) and LIWC2007 (Pennebaker et al., 2007). The latest release, LIWC2015 (Pennebaker et al., 2015), was introduced more as a new instrument than an updated variant of the old versions since, after a rigorous process of several years, some new categories were developed, others disappeared, while others kept their names but received an improved word composition. However, for example, in the study of Pennebaker et al. (2015), the correlations between the word frequencies counted with LIWC2015 and those obtained with LIWC2007 were very large—most of them were above 0.95—indicating that the new version tends to detect very similar linguistic patterns from one corpus to another as the old versions. This result might further suggest that the knowledge gained with previous LIWC tools could still be relevant to some extent for studies conducted with LIWC2015, despite some major differences in the composition of the dictionaries.

LIWC2015 and its predecessors have probably been the most preferred solution for automatic content analysis in social science research. According to our search performed at the end of May 2021, at that time, the Web of Science contained 736 records that included LIWC^*^ or *Linguistic Inquiry and Word Count* as keywords. Most authors opted for the generic term *LIWC* as a keyword. Therefore, estimating the number of papers in which LIWC2015 was a research tool would be impossible based on such a simplistic literature scan. However, we could notice that in 2016, one year after the release of LIWC2015, a significant increase in interest in LIWC dictionaries occurred, considering the 65 papers published in that year, which is almost double the number for 2015 (i.e., 36) and the highest number until then. In the following years, the ascending trend in using LIWC dictionaries for impactful research continued, peaking in 2020 with 124 papers indexed in Web of Science, after a similar number was reached in 2019 (i.e., 120). So far, scientists have used LIWC2015 for assessment in clinical psychology and psychotherapy (e.g., Wardecker et al., 2017; Huston et al., 2019), social psychology (Kwon et al., 2018; Klauke et al., 2019), personality psychology (e.g., Holtzman et al., 2019), education (e.g., Moore et al., 2019), health (e.g., Jordan et al., 2019), communication (Markowitz and Slovic, 2020), cultural psychology (e.g., Chen and Huang, 2019) or political science (Bond et al., 2017), to name a few application fields.

Besides LIWC2015, there are other closed-vocabulary resources to which psychologists can appeal. For instance, some well-known instruments are SentiWordNet (Baccianella et al., 2010), SentiStrength (Thelwall et al., 2010), and Affective Norms for English Words (ANEW; Bradley and Lang, 1999), which are specialized in emotional content, measuring valence (positivity, negativity, and neutrality), sentiment strength, or pleasure, arousal, and dominance, respectively. The *Lasswell dictionaries* and *Harvard psychosociological dictionaries* are other instruments designed to extract psychological-relevant meanings from language—for a presentation of them and many other closed-vocabulary approach tools, see Eichstaedt et al. (2020) and Neuendorf (2017). The list of dictionary-based tools to convert text into data is quite large and a thorough presentation in this regard would exceed the scope of this article. Nevertheless, the essence is that LIWC2015 has been particularly appealing and gained more popularity than other similar instruments due to several outstanding strengths and good timing of its development in the history of automatic language analysis, as we will briefly discuss further.

Thus, while General Inquirer (Stone et al., 1966), one of the first noteworthy and promising solutions to automatic psychological language analysis, suffered from several shortcomings—e.g., being difficult to manage, being expensive, and showing not enough satisfactory results compared to manual content analysis—that led to its decline, LIWC dictionaries rapidly took the stage (Boyd and Schwartz, 2021). The success of LIWC is explicable by its leverage on the rise of personal computing and the accumulating knowledge regarding the importance of the commonly ignored parts of speech in understanding human psychology (Boyd and Schwartz, 2021). Its growing notoriety continued until the present moment, especially with the new 2015 version, given its versatility in the research topics that it could address, good validity supported by evidence, and very intuitive and flexible software. All of these features are assets that are not cumulatively present in other closed-vocabulary approach tools. Moreover, in a preprint comparing five closed- and open-vocabulary approaches in terms of linguistic markers of gender, age, and personality (Eichstaedt et al., 2020), LIWC2015 led to better results than General Inquirer and DICTION.

LIWC2015, as opposed to similar tools, covers a wide range of content and grammar features that allow researchers to grasp both *what* and *how one* thinks. Specifically, the LIWC2015 dictionary contains approximately 90 categories referring to general text descriptors (e.g., the total number of words, the number of words per sentence), parts of speech (e.g., auxiliary verbs, adverbs), psychological constructs (e.g., emotions, drives), various contents (e.g., biological processes, relativity words), informal language (e.g., social media-specific words, swear words), and punctuation—for a comprehensive description of the LIWC2015 categories, see Pennebaker et al. (2015).

Likewise, when it comes to the structure of the LIWC2015 dictionary, an essential distinction arises between *content categories* (e.g., perceptual processes, drives, personal concerns, etc.) and *function word categories* (e.g., pronouns, articles, prepositions, conjunctions, negations, etc.). The former measure the discussed topics, whereas the latter help researchers understand how people approach their inner and outer experiences. In this regard, the brain area responsible for the content words is the temporal lobe, which deals with sensory input, emotions, and declarative memory (Chung and Pennebaker, 2018). Accordingly, content words indicate what information people associate and what preoccupies them. In contrast, function words are processed in the frontal lobe, signaling how one thinks about own person and relates to others (Chung and Pennebaker, 2018). Function words, which typically unfold as linguistic particles and have a high production rate, are precious because the speaker has little to no control over them and provide valuable insights into various psychosocial phenomena (Chung and Pennebaker, 2018; Boyd and Schwartz, 2021).

The composition of the original LIWC2015 dictionary has been established and evaluated from a psychometric perspective (Pennebaker et al., 2015), which is another significant strength that is hard to find in other similar instruments. The categories are displayed hierarchically—some lower-order ones define other superordinate ones—and the software provides outcomes for all of them. For instance, the *insight, causation, discrepancy, tentative, certainty*, and *differentiation* categories are all part of the *cognitive processes* category. Thus, a significant correlation between *cognitive processes* and another psychological construct could come from the association with one or more of the six subsumed categories. The LIWC2015 software reads various file formats and quickly generates results for all the categories in the dictionary. The outputs are easily transferable for data analysis without special preprocessing.

## LIWC2015 for Multilingual Analysis

LIWC2015 is already available in multiple languages, including Dutch (van Wissen and Boot, 2017), German (Meier et al., 2018), Ukrainian (Zasiekin et al., 2018), Brazilian Portuguese (Carvalho et al., 2019), and Romanian (Dudau and Sava, 2020). The number of translations would probably increase in the following years, considering that the previous LIWC dictionaries have been developed in over 10 languages and that conducting automatic language analysis has become one of the state-of-the-art approaches in psychology. Thus, LIWC2015 also provides researchers with the means to analyze texts directly in a targeted language or address intercultural questions associated with the psychological value of language. In other words, LIWC2015 creates a gripping research opportunity considering that, on the one hand, performing multilingual analysis has received growing interest, especially in the era of digital traces and globalization. On the other hand, most tools for automatic content analysis have been developed in English and are limiting in this regard, making multilingual analysis problematic (e.g., Balahur and Perea-Ortega, 2015).

At the same time, the translation and adaptation of the LIWC2015 dictionary from English to new languages to assure valid results in a multilingual setting pose significant challenges. Each language has many morphological, syntax, and semantic particularities, and specific phonetic symbols (for more thorough explanations about multiple languages, see Brown and Ogilvie, 2009). For instance, the Germanic languages are easily distinguishable from other Indo-European languages due to some clear-cut features, such as the so-called Grimm's Law, having a simplified verbal system consisted of two types of verbs—*weak* (regular) and *strong* (irregular), the last indicating the tense by an internal vowel change (e.g., ring, rang, rung)—or having a number of unique words in the vocabulary (e.g., Daniliuc, 2005). In the same line of thought, English, a West Germanic language, has no grammatical gender, making it different from most Indo-European languages (Swan, 2009). Likewise, English has well over 100 affixes in everyday use, with many of them, especially the suffixes (e.g., *-age, -ance, -ful, -ly, -en, -ify, -ize*), changing their word-class (Swan, 2009), which is a rule that does not apply identically in other languages. For example, in Romanian, one of the Romance languages in Eastern Europe, because such a rule does not exist for converting adjectives to adverbs, there is a substantial overlap between these two parts of speech.

Considering such between-language differences, every translation of LIWC2015 requires numerous decisions about which words and conjugations/declensions should be kept, dropped, or added. Likewise, the assignment into categories changes for some words due to semantic nuances that appear after translation. Such challenges occurred in the process of obtaining new LIWC dictionaries, as described in the papers dedicated to introducing the Spanish LIWC2001 (Ramírez-Esparza et al., 2007), French LIWC2007 (Piolat et al., 2011), Serbian LIWC2007 (Bjekić et al., 2012), Dutch LIWC2007 (Boot et al., 2017), or Romanian LIWC2015 (Dudau and Sava, 2020). For instance, in the Spanish, Romanian, and French dictionaries, the verbs required more conjugations than their English counterparts. Similarly, due to the grammar specificities of Serbian, a Slavic language, particular adaptations were necessary for *verbs*, and the *articles* category was not included in the Serbian LIWC2007. Likewise, in English, a word such as *blue* refers to both color and sadness/depression/gloominess. In contrast, it has only the first meaning in Spanish or Romanian, and after translation in these languages, it is not appropriate to be part of the *negative emotions* category like it is in the English dictionary. However, the second English meaning of the word *blue* is valid for the French translation.

Thus, translating LIWC2015 from English to other languages requires attention to both morphological and semantical aspects of every word in the dictionary, despite the category where that word belongs. However, the semantical dimension might be relatively easy to handle by using synonyms dictionaries in English and the target language, whereas the morphological component might be more challenging. In this regard, for instance, due to some meaning overlaps resulted in the translation language or some morphological particularities, it might not be possible to add a word stem followed by an asterisk^1^ every time such a construction appears in the original English dictionary or vice versa. Thus, instead of word stems, the appropriate conjugations/declensions are needed for the instrument to count the input words more accurately, and grammar knowledge and fine tunings are required in this respect.

Moreover, one might argue that some categories containing more content-focused words (e.g., anxiety, family, body, etc.) might end up being more similar to the original version in terms of derived word frequencies than those referring to the morphological particularities of each language (e.g., articles, prepositions, auxiliary verbs, etc.). For instance, English uses no article for generic reference, which is not the case with other European languages—e.g., English *music* becomes *die Musik* in German, *la musica* in Italian, and *la musique* in French (Swan, 2009). Likewise, English *music* as a generic reference, in Romanian, would be *muzica* with a definite article *-a* included in the word ending. Furthermore, Romanian *muzica* is identical to the no-article version (i.e., *muzica*) but without diacritics. Thus, some differences in the more linguistically specific grammar-related frequencies that the LIWC2015 software produces might vary from one dictionary version to another. For instance, previous research comparing the English LIWC2007 with the Serbian translation has already revealed such tendencies (Bjekić et al., 2014). However, overall, the semantical and morphological aspects relevant for creating new LIWC2015 dictionaries are rather intertwined for each word. Any assumptions regarding the differences/similarities at the category-level concerning the development of any new adaptation of LIWC2015 could only be exploratory and require research evidence.

In a nutshell, the discussion on translation and adaptation barriers and solutions is ample, differs from one language to another, and would not fit in a single non-theoretical article. Moreover, obtaining valid LIWC2015 dictionaries in other languages is not limited to the translation and adaptation process *per se*, since the frequency of the dictionary words found in the input data determines the language analysis outcomes. In this regard, the words that frequently appear in the input text weigh more than the others, while some words, although correctly translated and adapted to the new language, might not even count in the analysis. Thus, ideally, the new dictionary should include words with frequencies similar to those of the English LIWC2015 words, which is an unfeasible task. In this vein, developing a perfectly equivalent LIWC2015 adaptation is not possible^2^.

Therefore, several important questions arise: “To what extent does the translation of LIWC alter the original instrument—especially in the case of languages that differ significantly from English?”; “To what extent can we rely on different linguistic versions of LIWC as one instrument in cross-cultural studies to validly address research hypotheses concerning psychosocial phenomena?.”

Previous work has focused on validating a new LIWC translation against the English version. The usual procedure is to analyze corpora available in English and the targeted language and then confront the results. The correlational approach revealed high similarities between the English version of the dictionary and specific-language dictionaries, as happened with the Spanish LIWC2001 (Ramírez-Esparza et al., 2007), Serbian LIWC2007 (Bjekić et al., 2014), Dutch LIWC2015 (van Wissen and Boot, 2017), German LIWC2015 (Meier et al., 2018), etc. However, the authors who also applied the comparative approach, like Ramírez-Esparza et al. (2007), van Wissen and Boot (2017), or Meier et al. (2018), reported medium to large effect sizes for many mean-differences between the word percentages grasped with the English dictionary and those established with the new version. Moreover, we could not find any study about the equivalence between two or more LIWC2015 translations. In the same vein, as Lazarević et al. (2020) state, given that the majority of studies so far used the English LIWC2007 for data analysis, the extent to which those results show cross-linguistic and cross-cultural generalizability remains an open question. The relative patterns found in people from the same linguistic group should maintain, overall, leading to consistency across different-language groups, despite the between-language differences that might appear due to some syntax and pragmatics particularities of those languages. However, different-language replication studies are needed to sustain this assumption (Lazarević et al., 2020). Likewise, we argue that, in addition to addressing the cross-language generalizability in terms of intragroup patterns, there is a need for research concerning the validity of directly comparing the results of the psychological language analysis performed on different groups in cross-cultural studies.

## Research Goals and Hypotheses

The current study seeks to address the issue of using LIWC2015 translations as equivalent instruments across multiple languages. For this purpose, we placed the English, Dutch, Brazilian Portuguese, and Romanian LIWC2015 dictionaries in a multilingual setting, as explained in the *Method* section. Moreover, these four languages were appealing candidates for our research since their origins allow addressing supplementary exploratory hypotheses about potential between-dictionary differences and similarities.

Specifically, English and Dutch are West Germanic languages, whereas Brazilian Portuguese and Romanian are Romance languages. The Germanic languages presumably have descended from an ancestral well-differentiated language spoken by the people living around the North and Baltic Seas since the first century BCE. This old language has transformed into three dialects—the West, East, and North Germanic languages—and in the fifth century CE, the West Germanic tribes were the ones to bring into Britain a language that would later be known as English (Daniliuc, 2005). In contrast, the Romance languages have developed from Latin—probably its latest form, Vulgar Latin, of the fourth century CE—a language spoken around and beyond the Mediterranean Sea throughout the Roman Empire (Ponchon, 2005). Therefore, English and Dutch languages, on the one hand, and Brazilian Portugues and Romanian on the other, should share some particular features. We assume that these within-pair-specific features might have transferred in one form or another to the LIWC2015 dictionaries by the translation and adaptation process. The language analysis results generated with the LIWC2015 software on equivalent corpora might reflect this sort of dualism.

In another line of thought, consistent with previous work showing differences between LIWC2015 language versions, we argue that not considering the intraclass correlation in hierarchical data where the input language is a high-level variable would raise problems in approaching cross-cultural questions. In other words, if different-language dictionaries are not equivalent in the word counting results, using one dictionary or another is a variable that creates dependencies between observations. Namely, the input text for analysis is nested within language, which represents a two-level structure, with the language being a Level 2 variable. Due to such a Level 2 variable, the residuals correlate, leading to a violation of the independence assumption specific to linear models (Field, 2018). One solution to address the difficulties caused by the particularities of each language might be multilevel analysis, a linear model analysis in which the intercepts, slopes, or both, are set to vary across different contexts (e.g., van Herk and Fischer, 2017; Field, 2018). However, this solution might be problematic, especially when there are only a few language groups, given that the complexity of the multilevel models requires high sample sizes and many values for the context variable(s) to assure good statistical power. Furthermore, more straightforward solutions might also work.

Our goal for this paper is two-fold: (1) examining the equivalence issues of the English, Dutch, Brazilian Portuguese, and Romanian versions of LIWC2015 when disregarding language specificities; (2) testing the efficacy of an accessible corrective solution for the expected equivalence issues—i.e., within-language standardization. Additionally, we check whether excluding the grammar and informal language categories, which are more linguistically specific, improves the resemblance of the four LIWC2015 language versions.

To reach the first goal, we will use two approaches for data analysis: a machine learning-based strategy that would provide a view on the quantitative differences between the four dictionaries and a correlational-based method that would allow us to analyze the extent to which the four instruments acquire similar trends in homologous corpora and demonstrate “inter-rater” reliability. Given the previous studies that revealed strong correlations between the word percentages obtained with the English LIWC2015 and those computed with a LIWC2015 translation (e.g., van Wissen and Boot, 2017; Meier et al., 2018; Dudau and Sava, 2020), we expect our data to show evidence of equivalence between all dictionaries for the majority of the LIWC2015 categories in the correlational framework.

However, we argue that quantitative between-dictionary differences are likely to occur because of the specificities that characterize each language. Therefore, we assume that a supervised learning algorithm having as input the language analysis results obtained with the four dictionaries would accurately detect/predict the language of the input text in a multiclass classification problem. More precisely, we expect the model to achieve good classification accuracy—even though the cross-linguistic input is essentially the same—when the language particularities are not statistically handled. Namely, this would happen when the LIWC2015 percentages are standardized based on the whole-sample mean and standard deviation (i.e., grand mean standardization) as if the data were acquired with the same LIWC2015 instrument from a monolingual corpus.

Furthermore, any significant between-dictionary discrepancies explainable by the origins of the languages might come up from paired contrasts. Specifically, if the Romanian dictionary differs significantly from the English and Dutch versions, the Brazilian Portuguese dictionary should also be dissimilar. Likewise, suppose the distance between the Dutch and English dictionaries would be smaller than the difference between each one of them and the Romanian and Brazilian Portuguese tools. In that case, the similarity between the Romanian and the Brazilian Portuguese versions might also be higher than the similarity between them and the Dutch and English ones. In this vein, modern Dutch and English share many inflectional and derivational features (Simpson, 2009). For instance, in Dutch, the nouns and articles have no case distinctions, the pronouns have only two, and the plural form of the majority of nouns consists of adding either *-s* or *-en* at the word ending, as in English (e.g., Simpson, 2009). Moreover, as Durrell (2009) demonstrates, the similarity between Dutch and English—but also among Germanic languages—could be noticed in many lexical cognates, as it occurs, for example, in the English *house, red, I gave* vs. the very similar Dutch *huis, rood, ik gaf*. Thus, the linguistic closeness between Dutch and English might facilitate the adaptation of the LIWC2015 original dictionary to Dutch, and the contents and structure of the input texts might be more similar between these two languages. In contrast, both Romanian and Brazilian Portuguese are more complex morphologically speaking and show many differences when compared to Germanic languages. For instance, in Romanian and Brazilian Portuguese, both definite and indefinite articles have masculine and feminine forms, or the adjectives must agree in gender and number—in Romanian also in the case—with the nouns to which they refer. Likewise, in both languages, many words could transition easily to a different part of speech without changing their initial form (e.g., adjectives to adverbs), and the tenses of the verbs have more complicated structures than in English and Dutch (e.g., more variations in the auxiliary verbs)—for thorough explanations of the Brazilian Portuguese particularities, see Whitlam (2011), and for Romanian, refer to Cojocaru (2003).

In line with our second research goal, following the recommendations of Meier et al. (2018) for multilingual analyses, we test whether the language specificities that presumably bias the linguistic analysis results in cross-cultural research could be attenuated statistically. To this end, as a potential corrective strategy, we will convert the word percentages obtained with the LIWC2015 dictionaries into *z*-scores using the mean and standard deviation of each language subsample (i.e., within-language standardization). Then we will retrain the classification model and discuss its performance in distinguishing between the four languages. We expect our data to support the hypothesis that the four LIWC2015 translations produce similar results, even in this comparative framework, as an effect of within-language standardization.

To illustrate more clearly the rationale for assuming that within-language standardization is an adequate method for preparing the data for multilingual analysis, suppose we are interested in studying the relationship between *I-*statements and depression in a multilingual, cross-cultural study. In this example, the data structure is hierarchical: the first-person pronoun use extracted with LIWC2015 and the depression scores of people with different linguistic backgrounds are Level 1 variables; the participants' language (e.g., English, Spanish, Brazilian Portuguese, etc.) represents the Level 2 variable. Within-language standardization rescales the values of the Level 1 predictor (i.e., the amount of *I*-statements) around the mean of each linguistic cluster to where the participants belong. On the other hand, grand mean standardization rescales the values of the predictor around the mean of the entire cross-cultural sample. The scores resulted from both standardization strategies have the proprieties of a normal distribution with the mean equal to zero and a standard deviation of one. However, within-language standardization alters the participants' within-cluster rank order compared to the raw data—whereas grand mean standardization not. In other words, the advantage of within-language standardization is that it repositions the *I*-statement percentages by having as reference points the other cases in the same language cluster. The uncentered scores or those centered or standardized around the grand mean produce unclear relationships between the person/text-level variables (i.e., the amount of *I*-statements and depression)—because of the mixture of within- and between-language variations. In contrast, within-language standardization would provide outcomes uncorrelated with the language variable (Level 2), leading to a purer estimate of the Level 1 associations (for a more thorough discussion, see Enders and Tofighi, 2007).

In this vein, under grand mean standardization or no standardization, the raw data structure does not change, and the results would not validly reflect the participants' psycholinguistic features. Instead, the cluster variable (i.e., language), which includes between-dictionaries differences and language particularities, would bias the outcomes. Thus, within-language standardization appears as a promising solution for handling multilingual data even when the languages, and implicitly the word percentages determined with distinct LIWC2015 versions, differ. Having quantitative between-language differences in the research corpus might not be uncommon because every language has its syntax and pragmatics specificities. For instance, a text translated from English to a Slavic language tends to have 20% fewer function words (Lazarević et al., 2020). Likewise, there is a tendency to repeat the subject in Brazilian Portuguese, whereas in Spanish and European Portuguese, which otherwise are similar to Brazilian Portuguese, this habit does not exist (de Castilho, 2009). Therefore, testing the efficacy of a simple method to prepare the data for multilingual analysis, as the within-language standardization is, comes as a valuable endeavor.

## Method

### Sample and Procedure

To create a multilingual setting, we automatically saved from the official TED website (https://www.ted.com) the English, Dutch, Brazilian Portuguese, and Romanian transcripts of all-time conference talks held in English. TED hosts several types of events—e.g., TEDWomen, TEDSummit, independently organized local events, etc.—but we gathered only the transcripts of the talks labeled on the website as *TED Conference*. Then, we filtered out the talks for which the transcripts were not available in all languages. The data collection phase ended in July 2019. The final dataset comprised 7,012 transcripts (1,753 unique transcripts with counterparts in the four languages). Each corpus was analyzed with the LIWC2015 version of the corresponding language.

TED Conference is a notorious global event where prominent figures with various backgrounds (e.g., academics, scientists, philanthropists, environmentalists, artists, activists, etc.) meet two or more times a year to share meaningful, innovative ideas from their area of expertise. The talks have been recorded in audio-video format and made available online for free. The oldest records date from June 2006 and, considering that each meeting usually is a marathon of more than 50 talks, the repository accumulated to the present moment is quite large. Moreover, the TED website has a neat, well-organized structure, which facilitates automatic data collection. Thus, the multitude of TED data stored so far has become a valuable research resource, especially for language analysis, given that each talk has downloadable subtitles in multiple languages.

Furthermore, TED requests its volunteer translators meet several competency and quality standards (https://www.ted.com/participate/translate). For instance, the applicants for a TED translator position must be fluent in both source and target languages, pass an onboarding application quiz and learn all TED guidelines and best practices. The translated texts are reviewed by experienced volunteers who also provide feedback to the less-experienced community members. The TED Translators team also includes supervisors—volunteers with expert language skills and advanced knowledge in TED guidelines—who give supplementary advice and support. In terms of guidelines, TED requires the translators to choose informal over formal terms, preserve the speaker's tone and energy rather than do literal translations, find expressions similar to the original ones but natural in the target language, or favor modern over traditional terms, to name a few illustrative rules that shape the style and contents of the transcripts.

In this vein, we appreciate that the linguistic dataset gathered for the current study is balanced enough in terms of the formal-informal and personal-impersonal dimensions to provide a first pertinent view on the equivalence between the four LIWC2015 dictionaries. The cross-language translation quality and the size of the corpora also seem sufficient to approach our research goals and provide notable results, even though the details of the discussion that follows our findings might not be generalizable to other communication contexts.

### Measures

We used the English LIWC2015 (Pennebaker et al., 2015), Dutch LIWC2015 (van Wissen and Boot, 2017), Brazilian Portuguese LIWC2015 (Carvalho et al., 2019), and Romanian LIWC2015 (Dudau and Sava, 2020) to perform the automatic content analysis on the TED multilinguistic dataset. Our analysis narrowed down to 62 out of 93 LIWC2015 categories because we retained only the low-level grammar and psychological features in the hierarchical design of the tool. The higher-order features are typically composites of the lower-level ones and, therefore, including them would have brought no value in the machine learning model. We did not consider the punctuation variables since the software directly recognizes them—they were not independent entries in the dictionaries, and their counting should not vary between LIWC2015 versions. Moreover, we could not include four of the English LIWC2015 categories in our analyses—*analytical thinking, clout, authenticity*, and *emotional tone*—because the LIWC2015 authors decided not to disclose the composition of these variables. Thus, these four categories are secret components of the English dictionary and could be used only as part of the LIWC2015 original dictionary and software.

**The English LIWC2015** (Pennebaker et al., 2015) contains 6,549 labeled words, word stems, and emoticons selected based on a seven-stage process that involved teams of experts, estimates of the currency of the dictionary words, multiple human ratings, and internal consistency computations for each category. As we have also discussed in the *Introduction* section of this paper, the architecture of LIWC2015 is hierarchical, containing three general descriptor categories and 12 main high-order categories with sub-components referring to parts of speech, psychological constructs, personal concerns, markers of informal language, and punctuation.

**The Dutch LIWC2015** (van Wissen and Boot, 2017) emerged from an automatic translation and was improved by manual correction. The Dutch LIWC2015 performed well compared to the English LIWC2015, showing a mean correlation coefficient of 0.73 in a parallel-corpus approach. The tool has 13,440 entries assigned to the original LIWC2015 categories.

**The Brazilian Portuguese LIWC2015** (Carvalho et al., 2019) comprises 14,459 units distributed to 73 categories. The instrument outperformed the Brazilian Portuguese LIWC2007 (Balage Filho et al., 2013) in three studies.

**The Romanian LIWC2015** (Dudau and Sava, 2020) was built manually in one year and a half. The development protocol included extending the original lexicon with up to five Romanian synonyms per English word. Thus, the Romanian LIWC2015 contains 47,825 entries and 89 categories. The tool captured similar trends within a dataset of books compared to the English version and proved good criterion validity on a sample of posts from help-seeking forums.

### Analysis

To assess the equivalence of the four dictionaries, we employed two strategies. The first one was to reduce the dimensionality of the dataset to two principal components and use the variable loadings as inputs for a supervised learning model for determining the language of the transcripts, which is a multiclass classification problem. We used a support vector machine algorithm (SVM) with a linear kernel as a multiclass classifier. The rationale for restricting the SVM input to two dimensions was to obtain a graphical visualization of how the dictionaries positioned one relative to another based on the SVM performance.

This classification approach for comparing the four LIWC2015 dictionaries was implemented in two main research scenarios defined by what standardization method we used to prepare the data for multilingual analysis. As a general rule, by *standardization*, we mean the basic statistical procedure of subtracting the sample mean from each observation and dividing by the standard deviation. We used two standardization methods—grand mean standardization and within-language standardization—that are different only in terms of the reference parameters for rescaling the values: (1) whole-dataset mean and standard deviation; (2) subsample mean and standard deviation. Moreover, we considered two sets of LIWC2015 variables for each standardization scenario, as explained in the following paragraph. Thus, the machine learning-based approach was repeated four times.

First, we included all LIWC2015 categories and standardized the data without considering the language of the transcripts (grand mean standardization) before applying the principal component analysis (PCA). Second, we also used grand mean standardization but selected only the content categories in the dictionary (i.e., all LIWC2015 variables, without the *pronouns, other function words, other grammar*, and *informal language* ones). Third, we reintroduced all LIWC2015 categories, standardized the data at the level of each subsample (namely, the English, Dutch, Brazilian Portuguese, and Romanian transcripts, respectively), and proceeded with the PCA and SVM. Fourth, we selected only the LIWC2015 content categories, applied within-language standardization, and rerun the machine learning algorithm for the last time.

The second strategy for data analysis was to approach the between-dictionary equivalence in terms of correlational trends. In this regard, first, we computed Pearson's correlation coefficients for each pair of LIWC2015 dictionaries to check whether the four instruments tended to extract from the homologous transcripts linearly associated word percentages. In other words, we tested whether the dictionaries managed to grasp similar linguistic variations across the sample of TED transcripts.

Then, we determined the intraclass correlation coefficients (ICC), which, as opposed to Pearson's correlation coefficients, show the relationship not between different variables (i.e., *interclass correlation*) but between variables that have the same metric and variance—i.e., variable that are part of the same class (McGraw and Wong, 1996). In other words, ICC assesses the correlation between one or more measurements of the same targets, which represents a measure of *agreement* (Shrout and Fleiss, 1979; Portney, 2020). In our case, the *measurements* are the LIWC2015 dictionaries, and the *targets/objects of measurement* are the transcripts. Thus, the problem of between-dictionary equivalence reduces to asking whether the proportion of the total variance in our dataset attributable to the heterogeneity of the transcripts after controlling for other sources (e.g., the variance across the content analyses of the four dictionaries) is high enough to support between-dictionary agreement. To test this question, we applied a two-way mixed model for consistency and single scores.

The criteria of equivalence were: (1) poor accuracy in determining the language of the transcripts in the classification framework, as reflected by four parameters: sensitivity, specificity, F1-score, and area under the receiver operating characteristic curve (AUC); (2) high between-language Pearson's correlation coefficients for most categories; (3) good or excellent agreement as reflected by the ICCs. Specifically, to assess the between-dictionary equivalence based on the classification accuracy, we relied mainly on the AUCs and considered as a benchmark the 0.70 value, which is commonly used to discriminate between low and moderate accuracy (e.g., Akobeng, 2007)—a low accuracy (i.e., AUC < 0.70) meant that the dictionaries were equivalent.

Choosing a benchmark for the second criterion (i.e., Pearson's correlation coefficient) was more complicated in the absence of a generally approved standard for testing LIWC2015 dictionaries. Thus, the first hand reference value for appreciating whether the dictionaries produced similar outcomes was *r* > 0.50, which is the same equivalence metric used, for instance, by Meier et al. (2018) to validate the German adaptation of LIWC2015 or Boot et al. (2017) to test the equivalence of the Dutch and English LIWC2007 dictionaries. However, Pennebaker et al. (2015) appreciated that *r* < 0.80 was an indicator of a “low” correlation between the LIWC2007 and LIWC2015 English dictionaries. In the same vein, van Wissen and Boot (2017) obtained an average correlation of 0.73 between the machine-translated Dutch LIWC2015 and the English dictionary. On the other hand, the word percentages extracted with the LIWC tools tend to be context-sensitive (e.g., Mehl et al., 2012), and the coefficient obtained on other corpora than TED-talks might not provide a mathematically precise benchmark for our approach. Moreover, this is the first paper to address the equivalence of some LIWC2015 adaptations. For instance, we had no exact indicator on how strongly the Brazilian Portuguese and Dutch dictionaries should correlate. Therefore, in the current study, we considered Pearson's correlation coefficients higher than 0.50 were notable results toward between-dictionary equivalence. However, we ideally targeted values of at least 0.72, which, according to our computations, is the average correlation obtained by Meier et al. (2018) for the lower-level LIWC2015 features on a TED-talks corpus.

Regarding the ICC-based criterion, establishing what value should be decisive for assessing the between-dictionary equivalence is similar to choosing a benchmark for Pearson's correlation. However, to interpret our findings, we relied mainly on the usual recommendations for clinical research and aimed at ICCs of at least 0.75 as indicators of good between-dictionary agreement, with ICCs > 0.90 showing excellent reliability (Portney, 2020). According to the same standards, values between 0.50 and 0.75 suggest moderate reliability. Some authors might consider that ICCs slightly lower than 0.75/0.70 might also be appropriate in some contexts, especially in sociological and psychological studies or in the early phases of developing a new instrument (Portney, 2020).

## Results

### Preliminary Descriptive Analysis

The mean number of linguistic units counted with the LIWC2015 software ranged between 1,792.31 in the Romanian corpus and 1,980.93 for the English transcripts. The four dictionaries labeled roughly the same percentage of words, as shown in Table 1.

**Table 1:** Pearson's correlation coefficients for the word percentages obtained with the four LIWC2015 dictionaries.

	**Eng—Du**	**Eng—BP**	**Eng—Ro**	**Du—BP**	**Du—Ro**	**BP—Ro**
**Pronouns**						
I	0.99^**^	0.89^**^	0.88^**^	0.89^**^	0.88^**^	0.86^**^
We	0.97^**^	0.72^**^	0.75^**^	0.72^**^	0.75^**^	0.68^**^
You	0.93^**^	0.71^**^	0.65^**^	0.71^**^	0.64^**^	0.50^**^
She and he	0.74^**^	0.41^**^	0.30^**^	0.33^**^	0.33^**^	0.21^**^
They	0.83^**^	0.70^**^	0.60^**^	0.65^**^	0.58^**^	0.54^**^
Impersonal	0.73^**^	0.69^**^	0.50^**^	0.67^**^	0.55^**^	0.51^**^
**Other function words**						
Articles	0.82^**^	0.64^**^	0.45^**^	0.59^**^	0.43^**^	0.33^**^
Prepositions	0.77^**^	0.65^**^	0.64^**^	0.64^**^	0.61^**^	0.66^**^
Auxiliary verbs	0.74^**^	0.77^**^	0.18^**^	0.65^**^	0.20^**^	−0.01
Adverbs	0.58^**^	0.61^**^	0.34^**^	0.67^**^	0.31^**^	0.31^**^
Conjunctions	0.55^**^	0.69^**^	0.36^**^	0.52^**^	0.30^**^	0.30^**^
Negations	0.95^**^	0.86^**^	0.92^**^	0.86^**^	0.90^**^	0.80^**^
**Other grammar**						
Verbs	0.75^**^	0.72^**^	0.73^**^	0.64^**^	0.65^**^	0.64^**^
Adjectives	0.46^**^	0.68^**^	0.49^**^	0.37^**^	0.31^**^	0.44^**^
Comparisons	0.64^**^	0.68^**^	0.57^**^	0.54^**^	0.45^**^	0.45^**^
Interrogatives	0.66^**^	0.60^**^	0.67^**^	0.49^**^	0.55^**^	0.68^**^
Numbers	0.91^**^	0.72^**^	0.77^**^	0.68^**^	0.73^**^	0.81^**^
Quantifiers	0.61^**^	0.63^**^	0.66^**^	0.53^**^	0.53^**^	0.51^**^
**Affect**						
Positive	0.83^**^	0.64^**^	0.80^**^	0.60^**^	0.77^**^	0.65^**^
Negative	0.86^**^	0.83^**^	0.85^**^	0.75^**^	0.77^**^	0.76^**^
Anxiety	0.84^**^	0.82^**^	0.86^**^	0.74^**^	0.77^**^	0.77^**^
Anger	0.83^**^	0.82^**^	0.83^**^	0.74^**^	0.77^**^	0.76^**^
Sadness	0.69^**^	0.71^**^	0.60^**^	0.53^**^	0.44^**^	0.52^**^
**Social**						
Family	0.77^**^	0.88^**^	0.80^**^	0.70^**^	0.63^**^	0.81^**^
Friend	0.57^**^	0.56^**^	0.47^**^	0.52^**^	0.59^**^	0.53^**^
Female	0.81^**^	0.85^**^	0.67^**^	0.76^**^	0.58^**^	0.67^**^
Male	0.85^**^	0.70^**^	0.60^**^	0.65^**^	0.56^**^	0.51^**^
**Cognitive process**						
Insight	0.81^**^	0.81^**^	0.73^**^	0.70^**^	0.66^**^	0.65^**^
Causation	0.75^**^	0.58^**^	0.60^**^	0.53^**^	0.59^**^	0.52^**^
Discrepancy	0.66^**^	0.64^**^	0.72^**^	0.52^**^	0.59^**^	0.68^**^
Tentative	0.73^**^	0.63^**^	0.72^**^	0.50^**^	0.61^**^	0.62^**^
Certainty	0.60^**^	0.65^**^	0.47^**^	0.56^**^	0.50^**^	0.52^**^
Difference	0.77^**^	0.76^**^	0.69^**^	0.73^**^	0.65^**^	0.66^**^
**Perceptual processes**						
See	0.86^**^	0.80^**^	0.88^**^	0.75^**^	0.85^**^	0.77^**^
Hear	0.96^**^	0.82^**^	0.96^**^	0.83^**^	0.94^**^	0.79^**^
Feel	0.81^**^	0.60^**^	0.79^**^	0.59^**^	0.65^**^	0.54^**^
**Biological processes**						
Body	0.90^**^	0.90^**^	0.89^**^	0.87^**^	0.86^**^	0.87^**^
Health	0.91^**^	0.91^**^	0.94^**^	0.86^**^	0.88^**^	0.90^**^
Sexual	0.89^**^	0.85^**^	0.90^**^	0.87^**^	0.87^**^	0.86^**^
Ingest	0.93^**^	0.71^**^	0.91^**^	0.70^**^	0.87^**^	0.68^**^
**Drives**						
Affiliation	0.90^**^	0.74^**^	0.60^**^	0.68^**^	0.51^**^	0.65^**^
Achievement	0.76^**^	0.83^**^	0.66^**^	0.71^**^	0.59^**^	0.64^**^
Power	0.71^**^	0.75^**^	0.15^**^	0.70^**^	0.11^**^	0.12^**^
Reward	0.45^**^	0.35^**^	0.56^**^	0.28^**^	0.42^**^	0.45^**^
Risk	0.81^**^	0.55^**^	0.62^**^	0.51^**^	0.57^**^	0.41^**^
**Time orientation**						
Past	0.81^**^	0.88^**^	0.86^**^	0.77^**^	0.73^**^	0.81^**^
Present	0.74^**^	0.75^**^	0.78^**^	0.56^**^	0.64^**^	0.64^**^
Future	0.60^**^	0.67^**^	0.73^**^	0.53^**^	0.56^**^	0.63^**^
**Relativity**						
Motion	0.52^**^	0.62^**^	0.69^**^	0.39^**^	0.43^**^	0.53^**^
Space	0.80^**^	0.76^**^	0.81^**^	0.67^**^	0.76^**^	0.73^**^
Time	0.76^**^	0.64^**^	0.84^**^	0.66^**^	0.74^**^	0.67^**^
**Personal concerns**						
Work	0.81^**^	0.87^**^	0.87^**^	0.76^**^	0.75^**^	0.82^**^
Leisure	0.92^**^	0.65^**^	0.97^**^	0.62^**^	0.91^**^	0.64^**^
Home	0.88^**^	0.85^**^	0.83^**^	0.81^**^	0.78^**^	0.73^**^
Money	0.91^**^	0.89^**^	0.94^**^	0.84^**^	0.89^**^	0.89^**^
Religion	0.89^**^	0.90^**^	0.93^**^	0.83^**^	0.85^**^	0.87^**^
Death	0.88^**^	0.88^**^	0.93^**^	0.82^**^	0.86^**^	0.85^**^
**Informal language**						
Swear	0.40^**^	0.38^**^	0.28^**^	0.25^**^	0.24^**^	0.23^**^
Net speak	0.59^**^	0.14^**^	0.46^**^	0.32^**^	0.16^**^	0.11^**^
Agreement	0.79^**^	0.66^**^	0.78^**^	0.59^**^	0.69^**^	0.65^**^
Non-fluencies	0.50^**^	0.27^**^	0.76^**^	0.41^**^	0.48^**^	0.21^**^
Filler words	0.16^**^	0.12^**^	0.25^**^	0.14^**^	0.12^**^	0.01

*Eng, English; Du, Dutch; BP, Brazilian Portuguese; Ro, Romanian; N = 7,012; n = 1,753 transcripts per language;*

** *p < 0.01*.

### Classification Approach to Equivalence

We performed a principal component analysis in all classification scenarios to reduce the datasets from 62 to two independent variables for classification, as discussed in the *Analysis* subsection. Before the dimensionality reduction procedure, we randomly divided the 7,012 transcripts into two parts: 75% of the data points were assigned to the training subset and the rest to the test subset. Next, we used the datasets containing the variable loadings on the two principal components and the language labels for fitting and assessing the classification model.

Tables 2, 3 present the performance of the SVM models on the test subsets. The tables contain the results obtained across the two standardization scenarios when the input variables were all LIWC2015 categories and when only the psychological (i.e., content) features were included. The failure to distinguish between the source language of the transcripts as indicated by the accuracy parameters (e.g., AUC values below 0.70) could be considered indicative of between-language equivalence.

**Table 2:** Means (*M*) and standard deviations (*SD*) for the word percentages obtained with each LIWC2015 dictionary and the intraclass correlation coefficients (ICC) for the between-dictionary agreement.

	**Eng**		**Du**		**BP**		**Ro**		
	***M***	***SD***	***M***	***SD***	***M***	***SD***	***M***	***SD***	**ICC [95% CI]**
**Pronouns**									
I	2.78	2.21	2.81	2.27	1.87	1.63	1.17	1.12	0.83 [0.82, 0.85]
We	2.13	1.26	2.12	1.26	1.07	0.72	0.95	0.63	0.71 [0.69, 0.72]
You	1.87	1.25	1.82	1.27	1.59	0.93	1.05	0.82	0.69 [0.67, 0.70]
She and he	0.74	0.95	1.72	1.00	7.07	1.38	3.19	1.13	0.36 [0.33, 0.38]
They	1.12	0.72	1.45	0.85	2.58	0.94	1.18	0.56	0.63 [0.61, 0.65]
Impersonal	7.28	1.83	6.81	1.41	17.96	2.28	3.73	0.96	0.56 [0.54, 0.58]
**Other function words**									
Articles	7.40	1.49	9.59	1.82	12.64	1.82	4.27	0.98	0.54 [0.52, 0.56]
Prepositions	13.34	1.88	14.11	2.00	16.10	2.28	12.60	1.89	0.65 [0.63, 0.67]
Auxiliary verbs	8.93	1.66	7.20	1.41	6.95	1.35	4.45	1.13	0.45 [0.43, 0.48]
Adverbs	5.84	1.42	8.31	1.60	13.30	2.18	7.39	2.13	0.44 [0.41, 0.46]
Conjunctions	7.25	1.41	7.50	1.39	11.85	1.85	4.59	1.94	0.43 [0.40, 0.45]
Negations	1.25	0.62	1.34	0.62	1.54	0.64	1.65	0.84	0.86 [0.85, 0.87]
**Other grammar**									
Verbs	16.33	2.68	15.59	2.21	13.97	2.11	17.69	2.66	0.68 [0.66, 0.70]
Adjectives	4.17	1.10	6.84	1.33	4.09	1.01	7.71	1.51	0.43 [0.40, 0.45]
Comparisons	2.29	0.79	3.51	0.88	2.85	0.82	2.43	0.87	0.55 [0.53, 0.58]
Interrogatives	1.92	0.67	1.51	0.62	5.81	1.21	3.37	0.89	0.55 [0.53, 0.58]
Numbers	1.97	1.01	2.02	1.00	4.44	1.16	4.62	1.20	0.76 [0.75, 0.78]
Quantifiers	2.36	0.75	2.08	0.71	2.58	0.76	1.78	0.62	0.57 [0.55, 0.60]
**Affect**									
Positive	2.86	1.39	2.33	1.08	2.64	0.96	3.93	1.49	0.70 [0.68, 0.72]
Negative	1.27	0.87	1.12	0.68	1.53	0.89	2.37	1.20	0.76 [0.74, 0.77]
Anxiety	0.23	0.30	0.22	0.25	0.20	0.26	0.33	0.36	0.78 [0.77, 0.80]
Anger	0.32	0.38	0.26	0.29	0.30	0.36	0.58	0.55	0.74 [0.72, 0.75]
Sadness	0.25	0.30	0.29	0.27	0.29	0.28	0.57	0.44	0.54 [0.52, 0.56]
**Social**									
Family	0.28	0.44	0.43	0.64	0.31	0.46	0.46	0.58	0.73 [0.71, 0.75]
Friend	0.17	0.22	0.15	0.17	0.20	0.20	0.19	0.22	0.53 [0.51, 0.55]
Female	0.48	0.88	1.33	1.02	0.80	0.78	0.81	0.57	0.70 [0.68, 0.72]
Male	0.74	0.92	1.69	0.93	1.31	0.78	1.09	0.60	0.64 [0.62, 0.66]
**Cognitive process**									
Insight	2.47	0.92	2.96	0.88	2.20	0.85	3.09	1.16	0.71 [0.69, 0.72]
Causation	1.98	0.74	2.11	0.75	3.76	0.89	3.52	1.03	0.57 [0.55, 0.59]
Discrepancy	1.46	0.64	2.70	0.86	2.94	0.92	2.52	1.00	0.61 [0.59, 0.63]
Tentative	2.48	0.89	2.72	0.84	3.54	1.00	4.41	1.25	0.61 [0.59, 0.64]
Certainty	1.40	0.53	1.60	0.55	1.69	0.56	2.01	0.74	0.53 [0.50, 0.55]
Difference	3.05	0.90	3.11	0.92	4.07	1.04	3.81	1.07	0.70 [0.69, 0.72]
**Perceptual processes**									
See	1.25	0.91	1.06	0.75	1.38	0.85	1.33	0.96	0.81 [0.80, 0.83]
Hear	1.13	2.85	1.03	2.46	1.14	1.66	1.80	5.55	0.69 [0.67, 0.71]
Feel	0.40	0.38	0.35	0.33	0.54	0.42	0.45	0.41	0.65 [0.63, 0.67]
**Biological processes**									
Body	0.61	0.75	0.47	0.58	0.59	0.72	0.71	0.80	0.87 [0.86, 0.88]
Health	0.78	0.93	0.61	0.66	0.81	0.90	0.77	0.97	0.88 [0.87, 0.89]
Sexual	0.11	0.29	0.08	0.23	0.09	0.27	0.10	0.33	0.85 [0.84, 0.86]
Ingest	0.34	0.56	0.26	0.43	0.94	0.53	0.45	0.62	0.78 [0.77, 0.80]
**Drives**									
Affiliation	3.08	1.43	3.10	1.44	2.11	0.99	1.65	0.88	0.66 [0.64, 0.68]
Achievement	1.45	0.70	1.39	0.63	1.52	0.65	2.93	0.98	0.66 [0.64, 0.68]
Power	2.32	1.04	2.09	0.98	2.45	0.98	4.15	3.20	0.17 [0.15, 0.19]
Reward	1.19	0.58	0.83	0.58	2.49	0.73	1.03	0.54	0.40 [0.37, 0.42]
Risk	0.47	0.39	0.48	0.37	1.79	0.60	0.99	0.62	0.52 [0.49, 0.54]
**Time orientation**									
Past	3.82	1.88	5.47	1.77	2.91	1.55	8.16	2.54	0.77 [0.76, 0.79]
Present	11.17	2.64	12.72	2.10	8.78	1.84	10.76	2.42	0.68 [0.66, 0.70]
Future	1.11	0.56	2.29	0.79	0.83	0.50	0.97	0.62	0.59 [0.57, 0.61]
**Relativity**									
Motion	2.09	0.78	1.72	0.76	4.14	1.03	2.32	0.87	0.52 [0.50, 0.54]
Space	7.31	1.78	6.60	1.49	6.65	1.58	9.99	2.26	0.73 [0.71, 0.75]
Time	4.46	1.55	5.02	1.22	6.09	1.33	6.00	1.69	0.71 [0.69, 0.73]
**Personal concerns**									
Work	2.45	1.50	2.02	1.16	2.15	1.23	2.77	1.44	0.81 [0.79, 0.82]
Leisure	1.09	3.03	0.79	2.42	0.86	1.02	1.17	2.75	0.74 [0.72, 0.75]
Home	0.29	0.33	0.21	0.27	0.27	0.31	0.32	0.40	0.79 [0.78, 0.80]
Money	0.64	0.80	0.52	0.60	0.68	0.72	0.66	0.81	0.88 [0.87, 0.89]
Religion	0.19	0.42	0.17	0.34	0.21	0.41	0.22	0.46	0.87 [0.86, 0.88]
Death	0.18	0.32	0.15	0.25	0.18	0.29	0.20	0.35	0.86 [0.85, 0.87]
**Informal language**									
Swear	0.03	0.08	0.03	0.08	0.01	0.04	0.05	0.11	0.26 [0.24, 0.29]
Net speak	0.09	0.43	0.46	0.60	0.21	0.38	0.16	0.28	0.3 [0.27, 0.32]
Agreement	0.17	0.31	0.14	0.26	0.37	0.36	0.25	0.33	0.68 [0.66, 0.70]
Non-fluencies	0.20	0.24	0.03	0.11	0.19	0.22	0.07	0.20	0.39 [0.37, 0.42]
Filler words	0.01	0.05	1.71	1.15	0.65	0.44	0.01	0.03	0.04 [0.02, 0.06]

*Eng, English; Du, Dutch; BP, Brazilian Portuguese; Ro, Romanian; N = 7,012; n = 1,753 transcripts per language*.

**Table 3:** The composition of the transcripts based on the LIWC2015 tokenizer.

	**Word counts**		**Words per sentence**		**Dictionary words**	
**Language**	***M***	***SD***	***M***	***SD***	***M***	***SD***
English	1,980.93	999.15	25.71	54.83	86.62%	4.55%
Dutch	1,852.36	931.24	48.19	150.73	79.09%	4.69%
Brazilian Portuguese	1,915.69	957.49	52.31	97.51	79.59%	4.46%
Romanian	1,792.31	921.10	29.00	94.71	75.25%	5.12%

*Word counts = raw number of words; Dictionary words = the percentage of words in the analyzed text covered by the dictionary; N = 7,012; n = 1,753 transcripts per language*.

The accuracy parameters indicated that overall, the four LIWC2015 tools led to different results when the language specificities were disregarded (i.e., through grand mean standardization) than when they were considered (i.e., through within-language standardization). In the grand mean standardization scenario, there were little signs of between-dictionary equivalence. Thus, while we obtained pretty low discrimination indices between English and Dutch transcripts, the Brazilian Portuguese and Romanian transcripts dramatically distanced one from another and the other corpora. The confusion matrices for all analysis conditions are presented in Supplementary Tables 1, 2. Overall, under grand mean standardization, there was no significant change in the results after excluding grammar and informal language. Likewise, all AUCs obtained when the data were centered around the grand mean indicated an excellent discriminant performance (see the AUC_1_ column of **Table 5**), with one exception—the classifier showed poor accuracy in distinguishing between the English and Dutch transcripts (AUC = 0.65).

When we applied within-language standardization, the model had great difficulty identifying the correct language of the transcripts (see the AUC_2_ values in **Table 5**). For instance, when we considered all LIWC2015 categories, the SVM tended to misclassify many English, Dutch, and Brazilian Portuguese transcripts as Romanian (low specificity for the Romanian transcripts). Overall, these results suggest that when language specificities were taken into account, the four tools showed a high level of equivalence. The AUC values ranged between 0.47 and 0.53 when all LIWC2015 categories were inputs in the analysis and between 0.49 and 0.50 when we excluded the ones focused on grammar and informal language.

**Figure 1:**
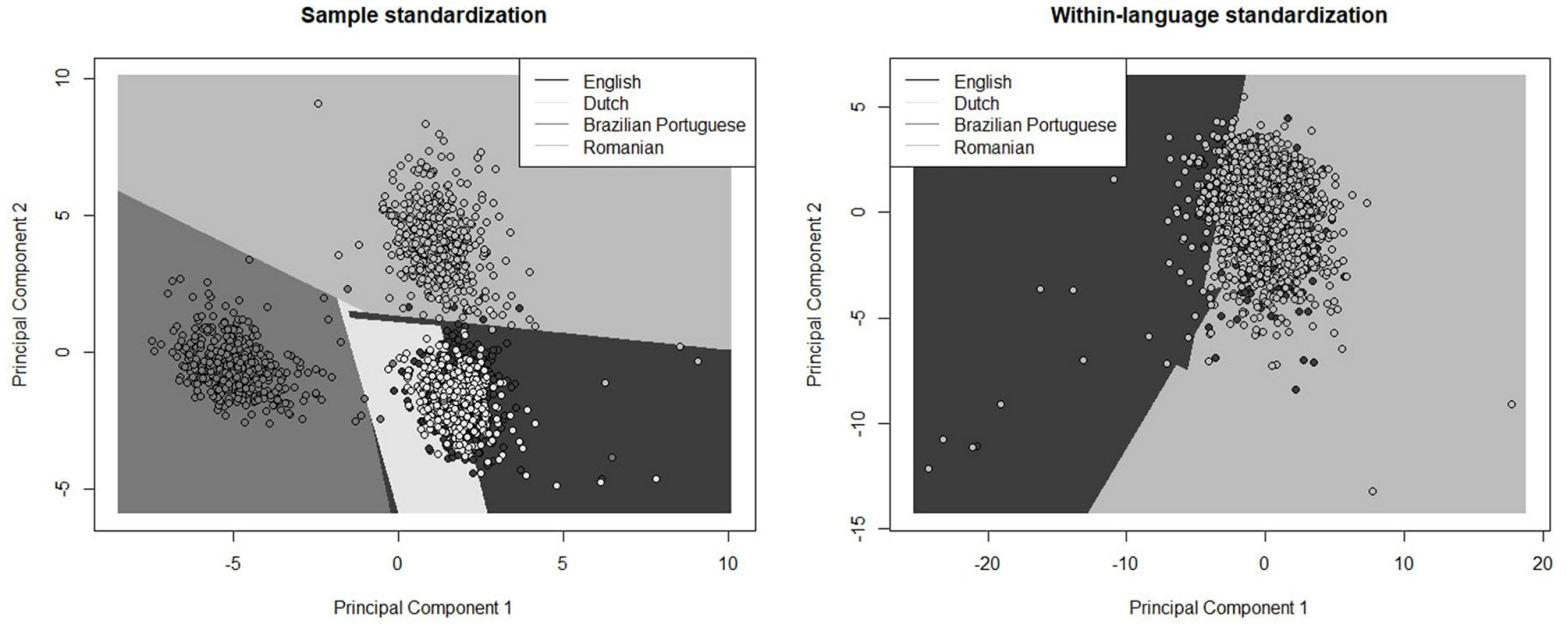
The performance of the SVM in estimating the language of the transcripts based on all LIWC2015 categories after grand mean standardization (image on the left) and within-language standardization (image on the right). The results were obtained on the test subset.

Figure 1 transparently depicts the above conclusions by presenting the position of each corpus relative to the other three when we used all LIWC2015 categories (additional visual representations are presented in Supplementary Figures 1, 2). The color of the dots represents the actual corpus to which the transcripts belong. The background color marks the class established by the SVM algorithm. Notably, when we standardized the data without considering the language specificities (i.e., grand mean standardization), a clear linear cut between the English and Dutch corpora was problematic—see the left side of Figure 1. In contrast, the Romanian and the Brazilian Portuguese corpora formed two homogenous and distanced clusters from the Dutch and English corpora and one from another. When we applied subsample standardization, the transcripts mixed considerably to a point where they could not group into clusters anymore, as depicted on the right side of Figure 1.

## Correlational Approach to Equivalence

The correlational framework for testing the between-dictionary equivalence consisted of computing two metrics—Pearson's correlation coefficient and ICC—reflecting slightly different perspectives, as explained in the *Analysis* subsection. Table 4 contains the Pearson's correlation results, while Table 5 shows the means and standard deviations recorded for each LIWC2015 category across the four languages, along with the ICCs for the inter-dictionary reliability. Of the two approaches, we will rely mainly on the ICCs in assessing whether the four instruments are equivalent. This type of analysis measures the agreement between multiple tools supposedly the same and is closer to the concept of multilingual analysis.

**Table 4:** The performance of the SVM in classifying the transcripts.

		**Sensitivity**		**Specificity**		**F1-score**	
**Input variables**	**Class**	**GMS**	**WLS**	**GMS**	**WLS**	**GMS**	**WLS**
**All LIWC2015 categories**	Eng	0.44	0.06	0.87	0.91	0.48	0.09
	Du	0.61	0.08	0.82	0.93	0.56	0.12
	BP	0.99	0.01	1	0.99	1	0.02
	Ro	0.99	0.87	0.99	0.17	0.98	0.40
**LIWC2015 content categories**	Eng	0.62	0.84	0.89	0.15	0.64	0.38
	Du	0.72	0.01	0.89	0.99	0.70	0.02
	BP	0.94	0.14	0.98	0.86	0.95	0.18
	Ro	0.88	0.00	0.95	1	0.87	–

*GMS, Grand mean standardization; WLS, Within-language standardization; The results were obtained on the test subset; Eng, English; Du, Dutch; BP, Brazilian Portuguese; Ro, Romanian; N = 1,752; n = 438 transcripts per language*.

**Table 5:** The AUC for the binary classifications.

**Input variables**	**Class_**1**_**	**Class_**2**_**	**AUC_**1**_**	**AUC_**2**_**
**All LIWC2015 categories**	English	Dutch	0.53	0.49
		Brazilian Portuguese	0.98	0.47
		Romanian	0.98	0.50
	Dutch	Brazilian Portuguese	0.99	0.48
		Romanian	0.99	0.52
	Brazilian Portuguese	Romanian	0.99	0.53
**LIWC2015 content categories**	English	Dutch	0.65	0.50
		Brazilian Portuguese	0.90	0.50
		Romanian	0.92	0.49
	Dutch	Brazilian Portuguese	0.96	0.50
		Romanian	0.94	0.50
	Brazilian Portuguese	Romanian	0.89	0.49

*AUC_1_, AUC in the grand mean standardization scenario; AUC_2_, AUC in the within-language standardization scenario; The results were obtained on the test subset; N = 1,752; n = 438 transcripts per language*.

The Pearson's correlation coefficients in Table 1 revealed that the four tools generated consistent results overall. According to the typical statistics guidelines, the effect sizes were large (i.e., *r* > 0.50) for 83.87% of the pairwise associations across languages (312 out of 372 pairs). Thus, in general, for a high number of LIWC2015 components, the variance in the word percentages computed with one dictionary explained at least 25% of the variability in the outcomes obtained with another dictionary for corresponding linguistic contents. The most troublesome features for multilingual analysis were several grammar categories (e.g., *third-person singular pronouns, auxiliary verbs, adverbs, conjugations, adjectives*), the *reward* category, and the *informal language* categories except *agreement*. On the other hand, 167 out of 372 pairwise associations attained the stricter criterion of *r* ≥ 0.72, most of them, 44, belonging to the Dutch–English pair, followed by the Romanian–English pair with 33 effect sizes this large. The LIWC2015 categories that showed very high equivalence (*r* ≥ 0.72) across all language pairs were mainly of the content-type ones (e.g., *negative emotions, see* and *hear* of the perceptual processes, *body, health, sexual, focus on past*, as well as the words related to *work, home, money, religion*, or *death*). Of the grammar-type categories, the *I*-statements and *negations* also demonstrated very strong associations across all language pairs. The *first-person plural pronouns* and the words related to *numbers* were also close to this performance.

According to the mean ICC value across all LIWC2015 categories, the four dictionaries showed moderate agreement (mean *ICC* = 0.63), with a lower consistency for the grammar and informal language categories (mean *ICC* = 0.54) than for the content-focused ones (mean *ICC* = 0.69). However, given the high number of LIWC2015 components and that we had more than two instruments, assessing the between-dictionary equivalence would require more fine-grained interpretations. Only 11 categories out of 62 did not have sufficient reliability for multilingual analysis (ICC < 0.50), at least not on our TED-talks dataset. Most of them were grammar categories—i.e., *she/he, auxiliary verbs, adverbs, conjunctions, adjectives*—indicating possible language specificities in the input texts, the composition of the dictionaries, or both. For instance, the mean percentages of *she/he* words and *adverbs* computed with the Brazilian Portuguese dictionary were significantly higher than the percentages detected with the other dictionaries, which means that the Brazilian Portuguese dictionary was probably the cause for the low ICCs on those two categories. Likewise, in terms of mean percentages, the Romanian dictionary showed the highest inconsistency with the other LIWC2015 tools and might have been the primary source of disagreement for *auxiliary verbs* and *adjectives*. The *informal language* categories demonstrated very low ICCs, too. The reason for this might have been the low variance across the transcripts—not necessarily some potential linguistic particularities—given the nature of the TED conferences (e.g., the chances for *swear words* to occur in this type of talks are meager). The poor reliability for the *power* and *reward* categories might have come from the differences in word coverage, lack of variance across transcripts, linguistic or cultural particularities that affected the construction of the dictionaries, or any other less evident reason.

On the other hand, 16 LIWC2015 variables revealed good between-dictionary agreement (i.e., ICC ≥ 0.75). Consistently with the Pearson's correlation results, they mainly were categories with a clear content focus: *negative emotions, anxiety, see*, all the *biological processes* categories, *focus on the past*, and all the *personal concerns* categories except *leisure*. Additionally, good ICCs were obtained for the *first-person singular pronouns, negations*, and *numbers*, which are probably easier to measure, in general, in all four languages. Notably, the *anger, family, space*, and *leisure* categories had good ICCs (i.e., ICC ≥ 0.75) as the upper bound of the confidence interval, and 11 other variables had near good ICCs (i.e., 0.70 ≤ ICC < 0.75) at the right end of the confidence interval. Among the last ones were three function words: *first-person plural pronouns, second-person pronouns*, and *verbs*. Thus, we might assume that on a different TED-talks sample, the four LIWC2015 dictionaries might achieve good reliability even on more linguistic variables. All the other undiscussed LIWC2015 categories reflected moderate between-dictionary agreement (i.e., 0.50 ≤ ICC < 0.75)—for more details, see Table 2.

## Discussion

In the current study, we aimed to assess the validity of LIWC2015 in a multilingual setting. Previous work has focused solely on the equivalence between a new LIWC language version and the original English dictionary, but testing how similar LIWC translations are one to each other has been overlooked. Multilingual analysis can enable exciting cross-cultural inquiries applicable to a wide range of research problems. However, it raises significant challenges due to the particularities of different languages that can easily affect the insights. Thus, a resounding question of how suitable is LIWC2015 for multilingual analysis arises. We addressed it in two ways: using a classification problem and a correlational framework of two types (i.e., computing Pearson's correlations and the ICCs for inter-dictionary reliability) on a large corpus of parallel texts in English, Dutch, Brazilian Portuguese, and Romanian.

The classification approach indicated that the Brazilian Portuguese and Romanian LIWC2015 departed significantly from the Dutch and English dictionaries when we did not apply within-language standardization. The results obtained with the Dutch LIWC2015 tended to mix with those extracted with the English LIWC2015, suggesting more similarity between the two tools. In this regard, Brazilian Portuguese and Romanian are Romance languages, whereas Dutch and English are Germanic languages. This fact might explain why the Brazilian Portuguese and Romanian classes distanced so much from the English-Dutch cluster. Modern Dutch is a typical Germanic language and, from a linguistics perspective, differs very little from modern English in terms of inflectional and derivational aspects (Simpson, 2009) and shares with it many lexical structures (Durrell, 2009).

Moreover, Dutch has simpler rules for constructing the verb tenses than the Romanian and Brazilian Portuguese. Likewise, Romanian and Brazilian Portuguese have in common some grammar peculiarities, such as more different words for the same parts of speech (e.g., articles, auxiliary verbs) or words that transition to different parts of speech without changing their initial form (for more thorough explanations concerning the grammatical specificities of the Brazilian Portuguese and Romanian, see Whitlam, 2011, and Cojocaru, 2003). However, the Brazilian Portuguese and Romanian tools also distinguished one from each other, suggesting a lack of equivalence from a direct comparison perspective, although the two languages have common origins (i.e., are Romance languages). In this regard, we should note that a more in-depth analysis of the influences that affected the development of these two languages suggests that comparing them based on their common origin is not so straightforward. Specifically, while being very conservative about some Romance features, the Romanian language has become very original in some aspects, given the Byzantine, Slavic, Greek, Hungarian, and French influences (Ponchon, 2005). In contrast, Brazilian Portuguese borrowed some features from the indigenous languages such as Tupi and those spoken by former African slaves (Bantu) while also keeping the Portuguese particularities acquired from influences that did not affect Romanian (Ponchon, 2005).

However, the classification performance only slightly worsened when we kept just the content categories as inputs, and the between-language patterns remained under the grand mean standardization. Specifically, the classification accuracy was still high with the same exceptions (i.e., the English–Dutch pair), indicating that the grammar and informal language particularities might not be the only cause for concern when multiple LIWC2015 translations are used for assessment. However, within-language standardization emerged as a viable solution to alleviate the between-language specificities that could threaten the validity of the quantitative comparisons in a multilingual setting.

The correlation analyses provided evidence to support the equivalence between the four dictionaries. The pairwise Pearson's correlation coefficients revealed that, overall, the English, Dutch, Brazilian Portuguese, and Romanian LIWC2015 tended to capture similar content variations from one transcript to another, as expected based on previous research concerning the similarity between the English LIWC2015 and different adaptations. There were a few grammatical categories that showed statistically significant but lower correlations. The ICC-based reliability analysis indicated that the four instruments tended to agree on most LIWC2015 categories. Specifically, ICCs revealed significant between-dictionary inconsistency (i.e., poor agreement) only for several grammar features, the *power* and *reward* categories, and *informal language*. This disagreement once again signaled potential language specificities in the input texts, the composition of the dictionaries, or both, and was attributable not necessarily to a general inconsistency across the four dictionaries.

For instance, the outcomes obtained with the Brazilian Portuguese LIWC2015 stood out from the other three languages in terms of third-person singular pronouns, which could explain the low ICC. Furthermore, this result might be consistent with the tendency to repeat the subject in Brazilian Portuguese, which probably creates different linguistic patterns in the usage of *she/he* words, leading to linguistic analysis results inconsistent with those produced with other dictionaries. The categories that reflected a good or almost good between-dictionary agreement referred to less linguistically specific function words (e.g., negations, numbers, and *I*-statements) or to clear psychological- or other-type contents (e.g., *negative emotions, biological processes*, or *personal concerns*). For approximately half of the categories, the inter-dictionary reliability was moderate. However, these lower ICCs might also be favorable enough to signal equivalence, depending on the benchmark used for interpreting the values. The cut-point that we used for good agreement (ICC ≥ 0.75) is specific to clinical research, where the diagnostic decisions usually have more serious implications than in some types of psychological research.

Together, the results of our classification and correlational approaches sustain the recommendation of standardizing or centering the data for each language subsample before performing a comparative analysis in a multilingual setting, as Meier et al. (2018) suggested. Studies that use monolingual data or treat multilingual data with statistical methods based on simple linear associations do not require particular data preprocessing since different LIWC2015 versions tend to grasp similar trends across data points.

However, performing within-language standardization is more appropriate for some research questions than for others. For instance, if someone is gathering bilingual data to address a Level 1 question (e.g., “Are narcissistic people more prone to use self-referenced language?”), within-language standardization is suitable to avoid the lack of equivalence between LIWC2015 tools. Conversely, such a corrective strategy would be less effective when the research aims at Level 2 questions (e.g., “Are individualistic cultures mirrored in the frequency of *I*-statements, after controlling for narcissism?”). Likewise, the multilevel approach could be used when both Level 1 and Level 2 predictors are part of the central research questions, mainly when text input is available from a significant number of languages.

### Limitations and Future Directions

The current study brings to the forefront an assessment issue important for psychological research but insufficiently explored. Along the way, we provided new evidence for the validity of LIWC2015 dictionaries since we also tested the equivalence between versions that have never been confronted before (i.e., the Dutch, Brazilian Portuguese, and Romanian LIWC2015). Moreover, our findings align with previous research and prove that within-language standardization is an efficient solution to attenuate the language particularities that could affect hypothesis testing in a multilingual setting. However, the present study contains several limitations that leave room for improvement and future quest.

One important shortcoming stems from previous research showing that the communication context could produce variation in the frequency of both content and function words, which could impact the equivalence statistics for different LIWC2015 dictionaries (e.g., Meier et al., 2018) or affect the association between linguistic features and other psychological variables (e.g., Mehl et al., 2012; Tackman et al., 2019). However, to test our hypotheses, we used only a corpus of TED Conference transcripts. Given the broad topic coverage and the engaging nature of these talks, combined with the scientific flavor that characterizes any TED material, we estimate that the linguistic data we analyzed in the current study is semi-formal and that the generalizability of our results might be limited to some degree.

In this regard, although TED transcripts form a vast, high-quality source of multilingual data, the context associated with the conference might alter the linguistic contents that the speakers choose for communication. Delivering a TED talk means trying to communicate inspiring, powerful ideas to a large audience of highly-educated thinkers while meeting the organizers' strict requirements. Naturally, designing a TED speech is different from producing language in any other setting (e.g., meetings with friends, discussions with coworkers, private monologs, social media posts, writing fiction books, etc.). Therefore, the language analysis results obtained on TED transcripts might change on other datasets, given that some words are more suitable in some contexts than in others and that some underlying psychological mechanisms might shape the verbal behavior. In the same line of thought, notably, in any closed-vocabulary approach analysis, some highly frequent words used in the input text determine the outcomes for each dictionary category and, implicitly, the between-dictionary equivalence statistics or the relations to other variables. Hence, our findings might not necessarily refer to each LIWC2015 tool as a whole but only to parts of the dictionary, depending on what words the TED speakers and translators used.

Likewise, the translation process of the TED talks might have been another potential source of bias related to our dataset. In this regard, the TED guidelines, among other recommendations, encourage translators not to perform a word-by-word translation but to choose similar expressions in the target language while also preserving “the tone and flow of the speaker's original talk” (https://www.ted.com/participate/translate/guidelines). Such instructions might increase the quality of the translations in terms of how representative is the translation language for native speakers. However, we might argue that, at the same time, they could leave room for subjective interpretations and personal biases in the translation process, which could further lead to artificial differences between the translations of the same transcripts. Although the study of Meier et al. (2021) showed that the TED translators managed to keep the gendered language styles of the speakers even when the speakers had a different gender than theirs, we could not rule out the possibility that our dataset was affected by other personal variables that could have made the transcripts not truly equivalent across languages.

Finally, the fact that Dutch is a Germanic language, the same as English, was not the only particular feature of the Dutch lexicon compared to the Brazilian Portuguese and Romanian tools. The development procedure was different: the Dutch LIWC2015 resulted from machine translation, which closely followed the composition of the original dictionary, whereas the other two were built manually. In this vein, for instance, through manual translation, the composition of the dictionary might gain sensitivity to the specificities of the target language, and the language analysis results might better reflect the psychological features of that language. Whether the translation method of the lexicon (manual vs. automatic) affects the equivalence of tools like LIWC2015 emerges as a valid question suggesting that our findings might require even more nuances. For instance, the high equivalence between the English and Dutch LIWC2015 and the lower agreement between the English and the other two dictionaries in our study could be explained by the fact that the Dutch tool was a more precise translation of the original version, not necessarily because Dutch and English are similar languages. Likewise, lower equivalence between the English dictionary and a new version like the Romanian one, which includes synonyms and grammar adaptations, does not directly reflect the quality of content analysis on that particular language.

As a final thought about future research directions regarding multilingual analysis, we notice that an interesting idea has started to take shape only recently in the literature: translating the input test to English (or another language) and then analyzing it with the original LIWC2015 or other available and valid adaptation instead of translating the dictionary in another language. Such an approach could raise reasonable doubts considering that the dataset could suffer severe changes due to translation. Thus, the verbal behavior *per se* could become inconsistent with the participants' psychological reality (e.g., some essential psychological words could disappear or vice versa). However, studies such as the one conducted by Araújo et al. (2020) on 14 tools have started to show promising results that such a strategy could work. Likewise, Boot (2021), using several LIWC versions, multiple machine translation engines, and replicating the results in different languages (i.e., Dutch, German, and Spanish), suggested that the machine-translation approach might be better than translating the dictionary, at least for some languages. Thus, we argue that the validity of using an automatic method for translating the input text and using the translation for language analysis is an exciting topic for future studies about multilingual analysis.

## Conclusion

LIWC2015 is a valuable tool for multilingual analysis. However, special care is needed when the aim is to compare or classify contents extracted with multiple LIWC2015 versions or to address person-level questions using data with a hierarchical structure. Disregarding language specificities might lead to biased results, given that between-group differences may occur due to the differences between LIWC translations and between languages themselves. A viable solution to this problem is employing multilevel analysis with language as the Level 2 variable. A competitive alternative, primarily when the research focuses on linking Level 1 variables, is to perform within-language standardization on the LIWC2015 data before investigating the main research questions. This practice reduces the challenges posed by the language particularities.

## Data Availability Statement

The datasets presented in this study can be found in online repositories. The names of the repository/repositories and accession number(s) can be found below: Open Science Framework (OSF), doi: 10.17605/OSF.IO/6TN9K.

## Author Contributions

DPD generated the original ideas, gathered the data, conducted the analyses, and wrote the first draft of all the article sections. FAS refined the original ideas, gave feedback, and helped DPD edit the final draft of the manuscript. All authors contributed to the article and approved the submitted version.

## Conflict of Interest

The authors declare that the research was conducted in the absence of any commercial or financial relationships that could be construed as a potential conflict of interest.

## Footnotes

^1^In the LIWC dictionaries, the asterisk is used for efficiency reasons to replace all strings that could follow a word stem.

^2^Throughout this article, we use the terms *equivalent/equivalence*, not in an absolute sense but to suggest good enough similarity in the outcomes acquired with different LIWC2015 versions.

## References

[B1] Akobeng A. K. (2007). Understanding diagnostic tests 3: receiver operating characteristic curves. Acta Paediatrica 96, 644–647. 10.1111/j.1651-2227.2006.00178.x 17376185

[B2] Araújo M. Pereira A. Benevenuto F. (2020). A comparative study of machine translation for multilingual sentence-level sentiment analysis. Inf. Sci. 512, 1078–1102. 10.1016/j.ins.2019.10.031

[B3] Baccianella S. Esuli A. Sebastiani F. (2010). SentiWordNet 3.0: an enhanced lexical resource for sentiment analysis and opinion mining, in Proceedings of the Seventh Conference on International Language Resources and Evaluation (LREC'10), 2200–2204.

[B4] Balage Filho P. P. Pardo T. A. Aluísio S. M. (2013). An evaluation of the Brazilian Portuguese LIWC dictionary for sentiment analysis, in Proceedings of the 9 *th* *Brazilian Symposium in Information and Human Language Technology, STIL* 215–219.

[B5] Balahur A. Perea-Ortega J. M. (2015). Sentiment analysis system adaptation for multilingual processing: the case of tweets. Inf. Process. Manage. 51, 547–556. 10.1016/j.ipm.2014.10.004

[B6] Bjekić J. Lazarević L. B. Živanović M. KneŽević G. (2014). Psychometric evaluation of the Serbian dictionary for automatic text analysis-LIWCser. Psihologija 47, 5–32. 10.2298/PSI1401005B

[B7] Bjekić J. Lazarević L. J. Erić M. Stojimirović E. Dokić T. (2012). Razvoj srpske verzije rečnika za automatsku analizu teksta (LIWCser). Psihološka IstraŽivanja 15, 85–110. 10.5937/PsIstra1201085B

[B8] Bond G. D. Holman R. D. Eggert J. A. L. Speller L. F. Garcia O. N. Mejia S. C.. (2017). Lyin'ted”, “Crooked Hillary”, and “Deceptive Donald”: language of lies in the 2016 US presidential debates. Appl. Cogn. Psychol. 31, 668–677. 10.1002/acp.3376

[B9] Boot P. (2021). Machine-Translated Texts as an Alternative to Translated Dictionaries for LIWC. Retrieved from https://osf.io/preprints/tsc36/ (accessed January 12, 2021).

[B10] Boot P. Zijlstra H. Geenen R. (2017). The Dutch translation of the Linguistic Inquiry and Word Count (LIWC) 2007 dictionary. Dutch J. Appl. Linguist. 6, 65–76. 10.1075/dujal.6.1.04boo

[B11] Boyd R. L. Schwartz H. A. (2021). Natural language analysis and the psychology of verbal behavior: the past, present, and future states of the field. J. Lang. Soc. Psychol. 40, 21–41. 10.1177/0261927X20967028 PMC8373026 34413563

[B12] Bradley M. M. Lang P. J. (1999). Affective Norms for English Words (ANEW): Instruction Manual and Affective Ratings. Technical Report C-1. Gainesville, FL: The Center for Research in Psychophysiology, University of Florida.

[B13] Brown K. Ogilvie S. (eds.). (2009). Concise Encyclopedia of Languages of the World. Oxford, UK: Elsevier.

[B14] Carvalho F. Rodrigues R. G. Santos G. Cruz P. Ferrari L. Guedes G. P. (2019). Evaluating the Brazilian Portuguese version of the 2015 LIWC Lexicon with sentiment analysis in social networks, in Anais do VIII Brazilian Workshop on Social Network Analysis and Mining (Porto Alegre: SBC), 24–34. 10.5753/brasnam.2019.6545

[B15] Chen C. Y. Huang T. R. (2019). Christians and Buddhists are comparably happy on twitter: a large-scale linguistic analysis of religious differences in social, cognitive, and emotional tendencies. Front. Psychol. 10:113. 10.3389/fpsyg.2019.00113 30792673 PMC6374623

[B16] Chung C. K. Pennebaker J. W. (2018). What do we know when we LIWC a person? text analysis as an assessment tool for traits, personal concerns and life stories, in The Sage Handbook of Personality and Individual Differences, eds Zeigler-Hill V. Shackelford T. K. (Thousand Oaks, CA: SAGE Publications Ltd.), 341–360. 10.4135/9781526451163.n16

[B17] Cojocaru D. (2003). Romanian Grammar. Durham, NC: Slavic and East European Language Research Center (SEELRC), Duke University.

[B18] Daniliuc L. (2005). Indo-European 2: Germanic languages, in Encyclopedia of Linguistics, ed Strazny P. (New York, NY: Fitzroy Dearborn), 512–515.

[B19] de Castilho A. T. (2009). Portuguese, in Concise Encyclopedia of Languages of the World, eds Brown K. Ogilvie S. (Oxford, UK: Elsevier), 883–885.

[B20] Dudau D. P. Sava F. A. (2020). The development and validation of the Romanian version of Linguistic Inquiry and Word Count 2015 (Ro-LIWC2015). Curr. Psychol. 10.1007/s12144-020-00872-4

[B21] Durrell M. (2009). Germanic languages, in Concise Encyclopedia of Languages of the World, eds Brown K. Ogilvie S. (Oxford, UK: Elsevier), 447–449.

[B22] Eichstaedt J. C. Kern M. L. Yaden D. B. Schwartz H. A. Giorgi S. Park G.. (2020). Closed-and open-vocabulary approaches to text analysis: a review, quantitative comparison, and recommendations. Psychol. Methods [Preprint]. 10.31234/osf.io/t52c6 34726465

[B23] Enders C. K. Tofighi D. (2007). Centering predictor variables in cross-sectional multilevel models: a new look at an old issue. Psychol. Methods 12, 121–138. 10.1037/1082-989X.12.2.121 17563168

[B24] Field A. (2018). Discovering Statistics Using IBM SPSS Statistics. Thousand Oaks, CA: SAGE Publications Ltd.

[B25] Holtzman N. S. Tackman A. M. Carey A. L. Brucks M. S. Küfner A. C. Deters F. G.. (2019). Linguistic markers of grandiose narcissism: aLIWC analysis of 15 samples. J. Lang. Soc. Psychol. 38, 773–786. 10.1177/0261927X19871084

[B26] Huston J. Meier S. Faith M. Reynolds A. (2019). Exploratory study of automated linguistic analysis for progress monitoring and outcome assessment. Couns. Psychother. Res. 19, 321–328. 10.1002/capr.12219

[B27] Jordan K. N. Pennebaker J. W. Petrie K. J. Dalbeth N. (2019). Googling gout: exploring perceptions about gout through a linguistic analysis of online search activities. Arthritis Care Res. 71, 419–426. 10.1002/acr.23598 29781577

[B28] Kern M. L. Park G. Eichstaedt J. C. Schwartz H. A. Sap M. Smith L. K.. (2016). Gaining insights from social media language: methodologies and challenges. Psychol. Methods 21, 507–525. 10.1037/met0000091 27505683

[B29] Klauke F. Müller-Frommeyer L. C. Kauffeld S. (2019). Writing about the silence: identifying the language of ostracism. J. Lang. Soc. Psychol. 39:0261927X1988459. 10.1177/0261927X19884599

[B30] Kwon J. Y. Bercovici H. L. Cunningham K. Varnum M. E. (2018). How will we react to the discovery of extraterrestrial life?. Front. Psychol. 8:2308. 10.3389/fpsyg.2017.02308 29367849 PMC5767786

[B31] Lazarević L. B. Bjekić J. Živanović M. KneŽević G. (2020). Ambulatory assessment of language use: evidence on the temporal stability of electronically activated recorder and stream of consciousness data. Behav. Res. Methods 52, 1817–1835. 10.3758/s13428-020-01361-z 32016918

[B32] Markowitz D. M. Slovic P. (2020). Communicating imperatives requires psychological closeness but creates psychological distance. J. Lang. Soc. Psychol. 39:0261927X2090281. 10.1177/0261927X20902816

[B33] McGraw K. O. Wong S. P. (1996). Forming inferences about some intraclass correlation coefficients. Psychol. Methods 1, 30–46. 10.1037/1082-989X.1.1.30

[B34] Mehl M. R. Robbins M. L. Holleran S. E. (2012). How taking a word for a word can be problematic: context-dependent linguistic markers of extraversion and neuroticism. J. Methods Measure. Soc. Sci. 3, 30–50. 10.2458/v3i2.16477

[B35] Meier T. Boyd R. L. Mehl M. R. Milek A. Pennebaker J. W. Martin M.. (2021). (Not) lost in translation: psychological adaptation occurs during speech translation. Soc. Psychol. Personal. Sci. 12, 131–142. 10.1177/1948550619899258

[B36] Meier T. Boyd R. L. Pennebaker J. W. Mehl M. R. Martin M. Wolf M.. (2018). “LIWC auf Deutsch”: The Development, Psychometrics, and Introduction of DE-LIWC2015. Retrieved from https://osf.io/tfqzc/ (accessed October 23, 2019). 10.31234/osf.io/uq8zt

[B37] Moore R. L. Oliver K. M. Wang C. (2019). Setting the pace: examining cognitive processing in MOOC discussion forums with automatic text analysis. Interact. Learn. Environ. 27, 655–669. 10.1080/10494820.2019.1610453

[B38] Neuendorf K. A. (2017). The Content Analysis Guidebook, 2nd Edn. Thousand Oaks, CA: SAGE Publications. 10.4135/9781071802878

[B39] Pennebaker J. W. Booth R. J. Francis M. E. (2007). Linguistic Inquiry and Word Count (LIWC): LIWC2007. Austin, TX: LIWC.net.

[B40] Pennebaker J. W. Boyd R. L. Jordan K. Blackburn K. (2015). The Development and Psychometric Properties of LIWC2015. Austin, TX: University of Texas at Austin.

[B41] Pennebaker J. W. Francis M. E. Booth R. J. (2001). Linguistic Inquiry and Word Count (LIWC): LIWC2001. Mahwah, NJ: Lawrence Erlbaum Associates.

[B42] Piolat A. Booth R. J. Chung C. K. Davids M. Pennebaker J. W. (2011). La version française du dictionnaire pour le LIWC: modalités de construction et exemples d'utilisation. Psychologie Française 56, 145–159. 10.1016/j.psfr.2011.07.002

[B43] Ponchon T. (2005). Indo-European 4: romance, in Encyclopedia of Linguistics, ed Strazny P. (New York, NY: Fitzroy Dearborn), 517–521.

[B44] Portney L. G. (2020). Foundations of Clinical Research: Applications to Evidence-Based Practice, 4th Edn. Philadelphia, PA: FA Davis.

[B45] Ramírez-Esparza N. Pennebaker J. W. Garcia A. F. Suriá R. (2007). La psicología del uso de las palabras: un programa de computadora que analiza textos en español. Revista Mexicana de Psicología 24, 85–99.

[B46] Shapiro G. Markoff J. (1997). A matter of definition, in Text Analysis for the Social Sciences: Methods for Drawing Statistical Inferences From Texts and Transcripts, eds Roberts C. W. (Mahwah, NJ: Lawrence Erlbaum Associates), 9–31. 10.4324/9781003064060-1

[B47] Shayaa S. Jaafar N. I. Bahri S. Sulaiman A. Wai P. S. Chung Y. W.. (2018). Sentiment analysis of big data: methods, applications, and open challenges. IEEE Access 6, 37807–37827. 10.1109/ACCESS.2018.2851311

[B48] Shrout P. E. Fleiss J. L. (1979). Intraclass correlations: uses in assessing rater reliability. Psychol. Bull. 86, 420–428. 10.1037/0033-2909.86.2.420 18839484

[B49] Simpson M. (2009). Dutch, in Concise Encyclopedia of Languages of the World, eds Brown K. Ogilvie S. (Oxford, UK: Elsevier), 307–311.

[B50] Stone P. J. Dunphy D. C. Smith M. S. Ogilvie D. M. (1966). The General Inquirer: A Computer Approach to Content Analysis. Cambridge, MA: MIT Press.

[B51] Swan M. (2009). English in the Present Day, in Concise Encyclopedia of Languages of the World, eds Brown K. Ogilvie S. (Oxford, UK: Elsevier), 327–334.

[B52] Tackman A. M. Sbarra D. A. Carey A. L. Donnellan M. B. Horn A. B. Holtzman N. S.. (2019). Depression, negative emotionality, and self-referential language: a multi-lab, multi-measure, and multi-language-task research synthesis. J. Personal. Soc. Psychol. 116, 817–834. 10.1037/pspp0000187 29504797

[B53] Thelwall M. Buckley K. Paltoglou G. Cai D. Kappas A. (2010). Sentiment strength detection in short informal text. J. Am. Soc. Inf. Sci. Technol. 61, 2544–2558. 10.1002/asi.21416

[B54] van Herk H. Fischer R. (2017). Multilevel cultural issues, in Cross Cultural Issues in Consumer Science and Consumer Psychology (Springer, Cham), 191–211. 10.1007/978-3-319-65091-3_11

[B55] van Wissen L. Boot P. (2017). An electronic translation of the LIWC Dictionary into Dutch, in Electronic lexicography in the 21st century: Proceedings of eLex 2017 conference (Brno: Lexical Computing), 703–715. Available online at: https://elex.link/elex2017/proceedings/eLex_2017_Proceedings.pdf

[B56] Wardecker B. M. Edelstein R. S. Quas J. A. Cordón I. M. Goodman G. S. (2017). Emotion language in trauma narratives is associated with better psychological adjustment among survivors of childhood sexual abuse. J. Lang. Soc. Psychol. 36, 628–653. 10.1177/0261927X17706940 29180832 PMC5701514

[B57] Whitlam J. (2011). Modern Brazilian Portuguese Grammar: A Practical Guide. New York, NY: Routledge. 10.4324/9780203843925

[B58] Zasiekin S. Bezuglova N. Hapon A. Matiushenko V. Podolska O. Zubchuk D. (2018). Psycholinguistic aspects of translating LIWC dictionary. East Eur. J. Psycholinguist. 5, 111–118. 10.29038/eejpl.2018.5.1.zas

