# Supplementary material for: Performing Multilingual Analysis With Linguistic Inquiry and Word Count 2015 (LIWC2015). An Equivalence Study of Four Languages
Source: Front Psychol. 2021 Jul 12;12:570568. doi: 10.3389/fpsyg.2021.570568 (PMC8311520; doi:10.3389/fpsyg.2021.570568)
Supplement: Supplementary file 1 [file Table_1.pdf]

Supplementary Material – Additional results

Supplementary Table 1

The confusion matrix of the SVM classifier for establishing the language of the transcripts based on all LIWC2015 categories.

		Predicted class			
Standardization	Actual class	English	Dutch	Brazilian Portuguese	Romanian
Sample level (Grand mean)	English	194	237	0	7
	Dutch	169	266	0	3
	Brazilian Portuguese	1	2	434	1
	Romanian	5	0	0	433
Subsample level (Within-language)	English	26	30	6	376
	Dutch	34	34	6	364
	Brazilian Portuguese	49	33	4	352
	Romanian	30	27	1	380

Note. The results were obtained on the test set; N = 1,752; n = 438 transcripts per language.
