# Supplementary material for: Performing Multilingual Analysis With Linguistic Inquiry and Word Count 2015 (LIWC2015). An Equivalence Study of Four Languages
Source: Front Psychol. 2021 Jul 12;12:570568. doi: 10.3389/fpsyg.2021.570568 (PMC8311520; doi:10.3389/fpsyg.2021.570568)
Supplement: Supplementary file 2 [file Table_2.pdf]

Supplementary Material – Additional results

Supplementary Table 2

The confusion matrix of the SVM classifier for establishing the language of the transcripts based on the content categories of LIWC2015.

		Predicted class			
Standardization	Actual class	English	Dutch	Brazilian Portuguese	Romanian
Sample level (Grand mean)	English	270	128	5	35
	Dutch	112	316	0	10
	Brazilian Portuguese	8	1	413	16
	Romanian	19	15	17	387
Subsample level (Within-language)	English	369	2	67	0
	Dutch	373	4	61	0
	Brazilian Portuguese	369	6	61	2
	Romanian	374	5	59	0

Note. The results were obtained on the test set; N = 1,752; n = 438 transcripts per language.
