# Supplementary figures and images for: Performing Multilingual Analysis With Linguistic Inquiry and Word Count 2015 (LIWC2015). An Equivalence Study of Four Languages
Source: Front Psychol. 2021 Jul 12;12:570568. doi: 10.3389/fpsyg.2021.570568 (PMC8311520; doi:10.3389/fpsyg.2021.570568)

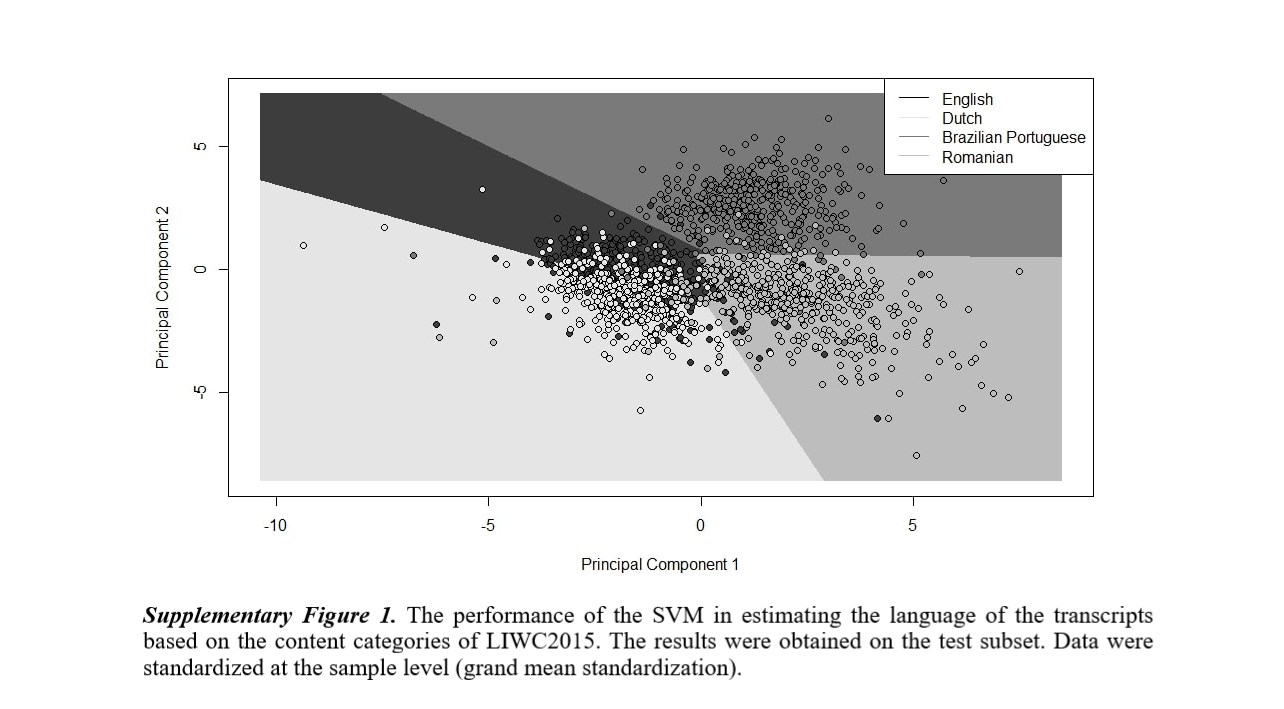

Supplement: Supplementary file 3 [file Image_1.png]

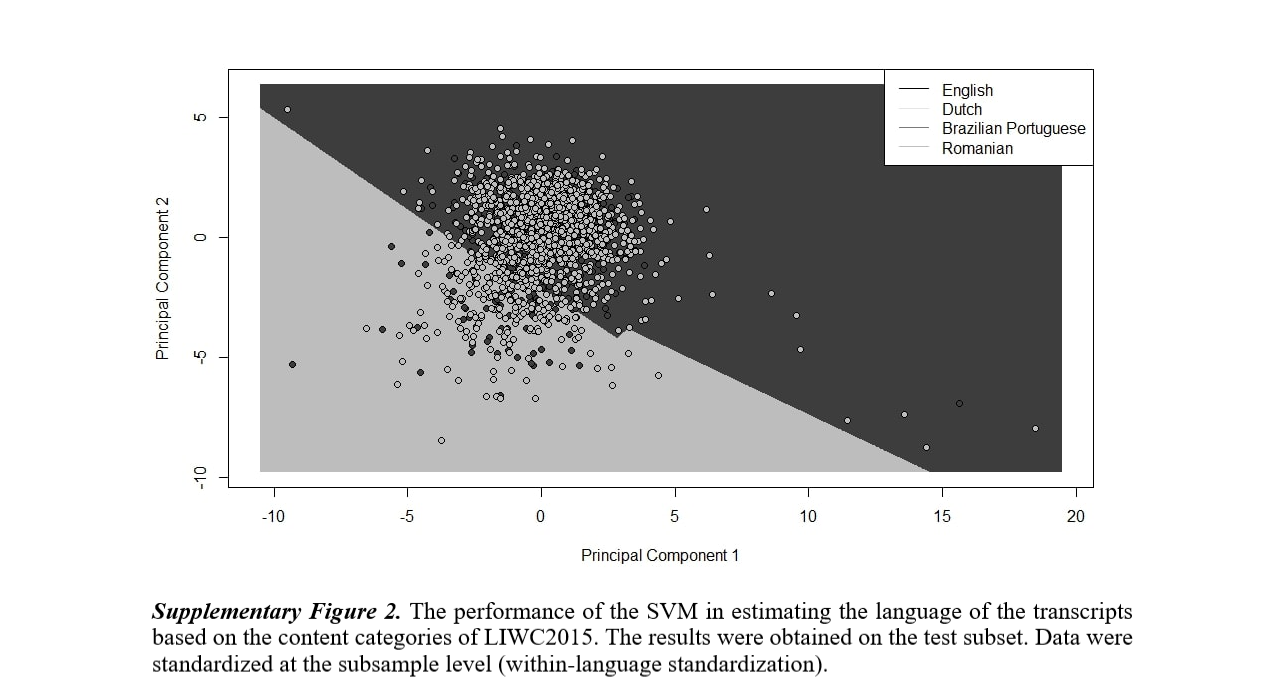

Supplement: Supplementary file 4 [file Image_2.png]
